# Supplementary figures and images for: Aerobic glycolysis supports hepatitis B virus protein synthesis through interaction between viral surface antigen and pyruvate kinase isoform M2
Source: PLoS Pathog. 2021 Mar 15;17(3):e1008866. doi: 10.1371/journal.ppat.1008866 (PMC8009439; doi:10.1371/journal.ppat.1008866)

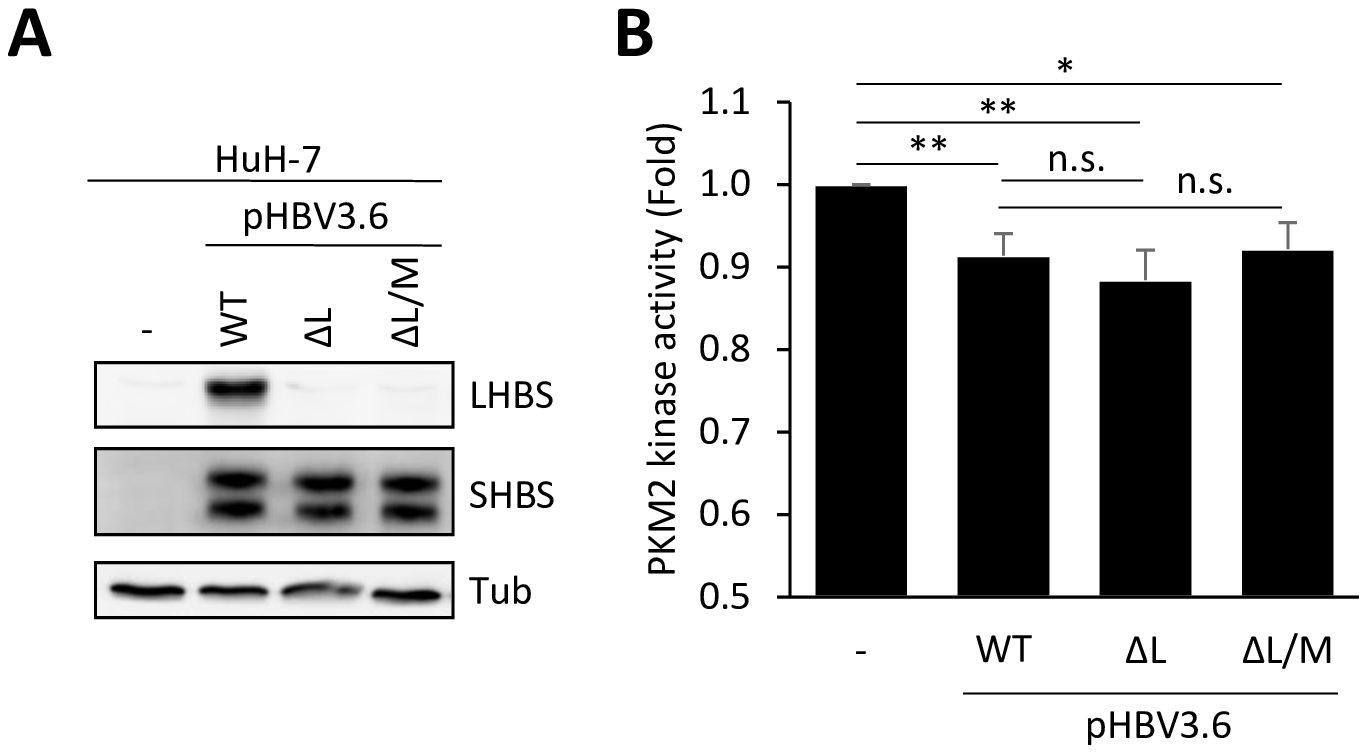

Supplement: S1 Fig — (A) HuH-7 cells were transiently transfected with pHBV3.6 wild type (WT), start codon mutant on PreS1 (ΔL) and double start codon mutant on PreS1/S2 (ΔL/M) for 48 hours. Expressions of LHBS and SHBS were detected by Western blotting. (B) PKM2 kinase activity was measured on transfected hepatocytes at 48 hours post-transfection. The quantitative result was a summary of five repeats and data were displayed as mean ± standard error. n.s., not significant. * p<0.05 and ** p<0.01 were calculated using Student’s t test. (TIF) [file ppat.1008866.s001.tif]

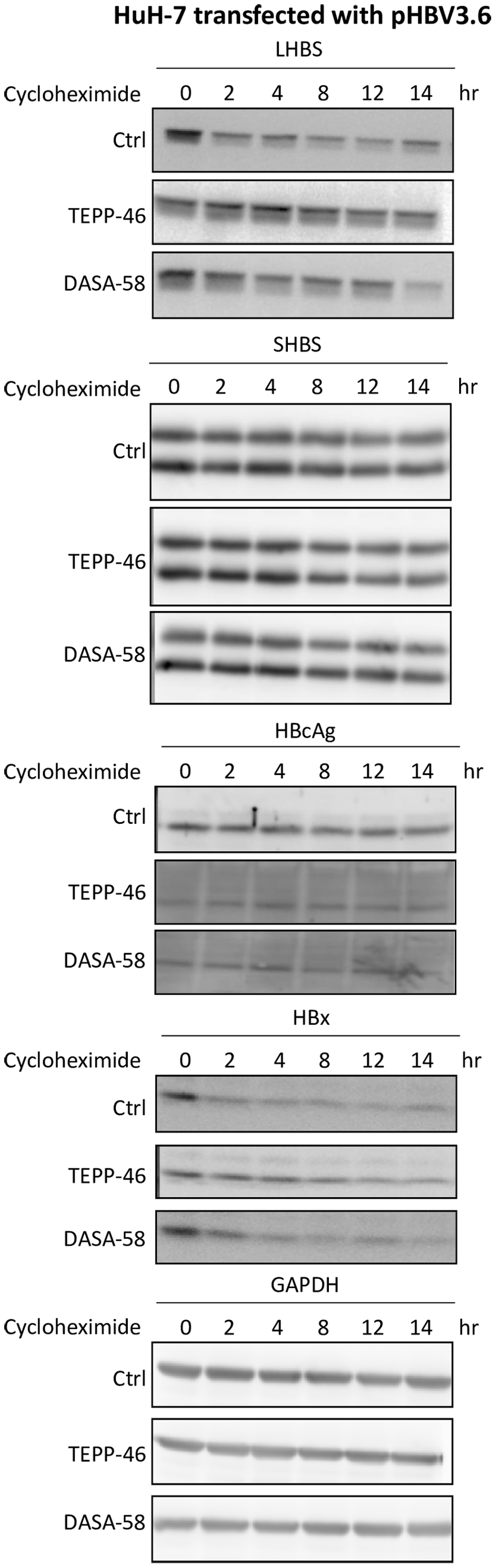

Supplement: S2 Fig — HuH-7 cells were transfected with pHBV3.6 for 24 hours and then treated simultaneously with 100 μg/ml cycloheximide, DMSO, 20 μM TEPP-46 or 50 μM DASA-58, as indicated. Total cell lysates were collected at indicated time points and subjected to western blotting using antibodies against LHBS/PreS1, SHBS, HBcAg, HBx, and GAPDH. Representative blots are shown. (TIF) [file ppat.1008866.s002.tif]

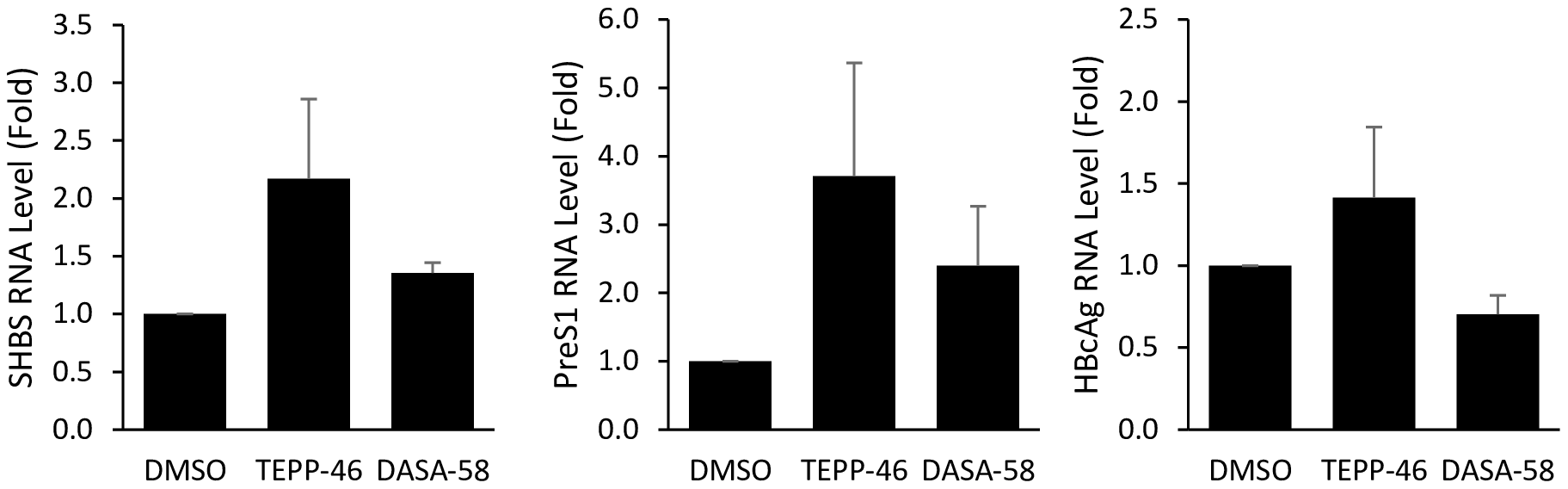

Supplement: S3 Fig — HuH-7 cells were transfected with pHBV3.6 for 24 hours and then treated with DMSO, 20 μΜ TEPP-46 or 50 μΜ DASA-58 for additional 24 hours. Total cell RNA was extracted and subjected to reverse transcription and quantitative PCR using primers specific for SHBS, PreS1 and HBcAg coding sequences. The quantitative results were a summary of three independent repeats and data were displayed as mean ± standard error. (TIF) [file ppat.1008866.s003.tif]
