# Supplementary material for: Aerobic glycolysis supports hepatitis B virus protein synthesis through interaction between viral surface antigen and pyruvate kinase isoform M2
Source: PLoS Pathog. 2021 Mar 15;17(3):e1008866. doi: 10.1371/journal.ppat.1008866 (PMC8009439; doi:10.1371/journal.ppat.1008866)
Supplement: S1 Text — (DOCX) [file ppat.1008866.s005.docx]

**Materials and methods**

**Plasmids**

The pHBV3.6 construct containing 1.2 copies of HBV genome was described previously [1]. Site-directed mutagenesis targeting start codons of PreS1 and PreS2 were performed to generate LHBS-deficient (ΔL) and LHBS/MHBS-deficient (ΔL/M) mutants, respectively, using serial In-Fusion cloning (Takara Bio). Primers used in this study were as follows: 5’-CTACAGCTTGGGAGGTTGGTCATCGAAA-3’, 5’-CCTCCCAAGCTGTAGCTCTTGTTCCCAA-3’ and 5’-CAAAGGCTTGGGGACGAATCTTTCTGTTC -3’, 5’-GTCCCCAAGCCTTTGCGAGGTTTCGATG-3’ for ΔL; further mutated PreS2 region using 5’-TCAGGCCTTGCAGTGGAATTCCACTGCC-3’ and 5’-CACTGCAAGGCCTGAGGATGACTGTCTC-3’ to generate ΔL/M.

**Reverse transcription PCR and real time PCR**

Total RNA from HuH-7 cells transfected with pHBV3.6 was isolated using REzol C&T (PROtech) and removal of genomic DNA by DNase I (New England BioLabs), followed the manufacturer's instructions. Approximately 2.5 μg total RNA was reverse transcribed into cDNA with SuperScript IV VILO Master Mix (Invitrogen), followed by quantitative PCR with 2x SensiFAST SYBR Lo-ROX Mix (BIOLINE) at 95°C for 5 seconds and 40 cycles of 95°C for 15 seconds and 60°C for 25 seconds on AriaMx Real-Time PCR System (Agilent Technologies). Primers used in this study were as follows: 5’-GTGTCTGCGGCGTTTTATCA-3’ and 5’-GACAAACGGGCAACATACCTT-3’ for SHBS; 5’-CCCGATCATCAGTTGGACCC-3’ and 5’-TGGTCAATATGCCCTGAGCC-3’ for PreS1; 5’-TTCCGGAAACTACTGTTGTTAGAC-3’ and 5’-ATTGAGATTCCCGAGATTGAGA-3’ for HBcAg; 5’-CATTGCTGACAGGATGCAGAAGG-3’ and 5’-TGCTGGAAGGTGGACAGTGAGG-3’ for actin, respectively.

Reference

1. Yuh CH, Chang YL, Ting LP. Transcriptional regulation of precore and pregenomic RNAs of hepatitis B virus. J Virol. 1992;66(7):4073-84. Epub 1992/07/01. doi: 10.1128/JVI.66.7.4073-4084.1992. PubMed PMID: 1602534; PubMed Central PMCID: PMCPMC241210.
