# Supplementary material for: Aerobic glycolysis supports hepatitis B virus protein synthesis through interaction between viral surface antigen and pyruvate kinase isoform M2
Source: PLoS Pathog. 2021 Mar 15;17(3):e1008866. doi: 10.1371/journal.ppat.1008866 (PMC8009439; doi:10.1371/journal.ppat.1008866)
Supplement: Attachment — Submitted filename: Point-by-point response-0125.docx [file ppat.1008866.s006.docx]

Reviewer's Responses to Questions

**Part I - Summary**
Please use this section to discuss strengths/weaknesses of study, novelty/significance, general execution and scholarship.

**We would like to thank all reviewers again for their positive feedback and appreciate the reviewers’ understanding of the challenges in our revision experiments, especially when COVID-19 significantly impacted the shipment of our research materials. In response to questions raised by the reviewers, we have performed new experiments such as to work with the infectious model system using HepG2-NTCP, new immunoprecipitations, and made corresponding changes in the revised manuscript. With regard to the use of HepG2-NTCP-C4 cells for supporting HBV infection, Dr. Koichi Watashi is now added as a new co-author (with written agreement of all authors provided to the editorial office). The major concerns of each reviewer are summarized below and are further detailed in the point-per-point response in Part II.**

Reviewer #1: This is a well written manuscript by Y-H Wu and colleagues.
Introduction and Discussion sections are particularly informative.
The initial hypothesis that HBV governs intracellular biosynthesis in infected cells to its advantage is interesting because it may help understand better the biology of HBV including the process of oncogenesis. For this latter reason, the authors chose to select the L HBV env protein for the viral target of choice.
An affinity purification experiment was first conducted to identify L-binding partners using a L-expressing cell line previously developed in the laboratory. Unfortunately, there are no details provided on either the level of L expression in this cell line, the pulldown experiment and the identified interactants other than pyruvate kinase isoform M2 (PKM2).
Was PKM2 the major binder? Because the initial pulldown was carried out in a somewhat artificial system (a tagged-L protein overexpressed in a non-hepatocyte cell-line), PKM2-L binding should be confirmed in HBV infected cells (PHH or NTCP-expressing cell lines).
Experiments reported in Fig.2 show that the C-terminus of PKM2 binds to the S domain of L HBV env. This suggests that S env protein would probably be the best binder to PKM2. Furthermore, S HBV env is, by far, the most abundant HBV protein in infected cells and should have logically been tested.
It is unclear to this reviewer whether the SNAP-L protein used as a bait in the pulldown experiments is myristoylated, and why the profiles of L, M and S proteins in western blot analysis (Fig.2B and D) are not conventional, for both size and glycosylation state. These observations are further indications that experiments should indeed be conducted in the context of an in vitro infection of HBV susceptible cells.
As the PKM2 protein activity is correlated to its degree of polymerization, the authors sought to evaluate the effect of L on PKM2 dimer and tetramer formation (Fig.3 and 4). The results presented in Fig.3B, 4B and 4E are not convincing, for lacking precise quantification and for ignoring that the levels of monomers appear to vary in parallel with dimers and tetramers Fig.4B and 4E.
The authors used TEPP-46 and DASA-58 drugs as known activator of PKM2 to treated HBV expressing Huh-7 cells. The experiment is not precisely described, but the results presented in Fig.6 show only a modest effect of the treatment on extra-cellular HBsAg (SVPs essentially) and HBeAg.
Overall the experiments reported in this manuscript lack information on statistical analysis and precise quantification. Measurement of western blot signals by digital imaging systems is not sufficiently precise.
Furthermore, it is not demonstrated that biosynthesis of HBV proteins, per se, is affected by aerobic glycolysis. What about protein degradation? And what about HBV DNA replication and virions production?
The conclusion of the study that L-HBV env protein would induce a metabolic switch (increase of glucose consumption and lactate production) to favor virus production would require that a study be conducted on in vitro infected cells, using more precise quantification tools. The statement in the Author Summary paragraph: “We show that metabolic switch not only favors biosynthesis of HBV but also provokes hepatocarcinogenesis” is not supported by the data.

We thank the reviewer’s comments and suggestions. In the revised manuscript, we have performed new experiments to strengthen our conclusion and proved that the metabolic switch induced by LHBS protein was also evident in an infectious model using HepG2-NTCP-C4 (Fig 3A-C, 6E). In addition, we performed new immunoprecipitation experiments to demonstrate the molecular interplay between PKM2 and native viral envelope proteins (Fig 2A). An additional list of interacting partners identified by mass spectrometry is included in the supplementary new S1 Table. Finally, we investigated RNA synthesis and stability of viral proteins upon treatment with PKM2 activators. We showed that aerobic glycolysis did not significantly affect viral RNA transcription and viral protein stability (S2 Fig and S3 Fig). As byproducts in the pentose phosphate pathway are the building blocks of de novo protein synthesis in the cell (Fig 6D), our results suggest that aerobic glycolysis is likely to provoke de novo protein synthesis of HBV viral products upon the activation of PKM2. Finally, we have provided information about the statistical analysis used in the results and detailed descriptions for protein quantitation.

Detailed point-by-point response and descriptions of our new results for addressing major issues are provided in Part II.

Reviewer #2: The authors used an affinity purification screen to identify host factors that interact with The HBV large surface antigen (LHBS or L). This effort lead to the identification of the cellular pyruvate kinase isoform M2 (PKM2), a key regulator of glucose metabolism, as a L binding partner. They then showed that the expression of LHBS affected oligomerization of PKM2 in hepatocytes, and increased glucose consumption and lactate production, i.e., aerobic glycolysis. Restoring PKM2 activity by chemical activators, TEPP-46 or DASA-58, reduced the levels of viral surface and core antigens. Reduction of glycolysis by culturing cells in low-glucose condition or treatment with 2-deoxyglucose also decreased viral surface antigen levels. PKM2 activation by TEPP-46 suppressed proliferation of LHBS-positive cells in 3-dimensional agarose culture, without showing any
effect on the traditional 2-dimensional cell culture. The authors concluded that HBV-induced metabolic switch may support biosynthesis of HBV in hepatocytes. In addition, aerobic glycolysis is likely essential for LHBS mediated oncogenesis.

Whereas some of the authors’ results are tantalizing and potentially significant in understanding HBV-host interactions in HBV replication and pathogenesis, the results are subject to alternative interpretations and more rigorous studies in more physiologically relevant systems are needed to verify the observations.

We thank the reviewer for the comments and suggestions. We have performed new experiments to address the issue regarding whether SHBS interacts with PKM2 (Fig 2A). In addition, we examined the influence of HBV on modulating PKM2 activity in an infectious model using HepG2-NTCP-C4. In the revised manuscript, we provided new evidence that PKM2 activity was reduced upon HBV infection (Fig 3C), and restoration of PKM2 activity suppressed the production of HBV virions of HBV-infected HepG2-NTCP-C4 (Fig 6E). Finally, we agree with the reviewer that more rigorous experiments are needed to draw the conclusion for the impact of HBS-PKM2 interaction on hepatocarcinogenesis. In the revised manuscript, we have revised our manuscript as appropriate. Detailed point-by-point response and descriptions of our new results for addressing major issues are provided in Part II.

Reviewer #3: Yi-Hsuan Wu and colleagues report in this manuscript that HBV large envelope protein LHBs interacts with pyruvate kinase isoform M2 (PKM2) and inhibits its enzymatic activity, which consequentially induces metabolic switch from oxidative phosphorylation to aerobic glycolysis, with increased glucose consumption and lactate production in hepatocytes. The authors claimed that the virus-induced metabolic switch not only favors the replication of HBV but also provokes hepatocarcinogenesis. Overall, the virus-induced metabolic reprogram is an important, but under-investigated area in HBV pathogenesis. The authors made some interesting findings, which require further mechanistic analysis and validation in HBV infected human hepatocytes.

We thank the reviewer’s comments. We have provided major evidence to demonstrate that PKM2 activity is modulated upon HBV infection in HepG2-NTCP-C4 cells (Fig 3C). In addition, we provide more evidence to support the molecular interaction between viral envelope proteins and PKM2 (Fig 3G and 3H), which was required for triggering aerobic glycolysis and thereby supporting viral biosynthesis through offering molecular building blocks from pentose-phosphate pathways (Fig 6D). We believe these new data provide strong support for the importance of metabolic switch in the production of HBV. Detailed point-by-point response accompanying new data support are provided in the below Part II.

**Part II – Major Issues: Key Experiments Required for Acceptance**
Please use this section to detail the key new experiments or modifications of existing experiments that should be absolutely required to validate study conclusions.

Generally, there should be no more than 3 such required experiments or major modifications for a "Major Revision" recommendation. If more than 3 experiments are necessary to validate the study conclusions, then you are encouraged to recommend "Reject".

Reviewer #1: Pyruvate kinase isoform M2 (PKM2) a key regulator of glucose metabolism was identified. PKM2-L HBV env binding should be confirmed in HBV infected cells.
Because the S domain of L HBV env has been identified as responsible for binding to PKM2, S env protein should be tested.
Overall the experiments lack precise quantification and information on statistical analysis.
Quantification of western blot signals by digital imaging systems is sufficiently precise.
It is claimed that biosynthesis of HBV proteins, per se, is affected by aerobic glycolysis. Protein stability should be evaluated. There is no analysis of HBV DNA and virions levels.

1. We thank the reviewer’s comments and suggestions. As suggested by the reviewer, we tested interaction between PKM2 and HBV surface antigens in an infectious model using HepG2-NTCP-C4 cells. The infectious HBV virions were collected from the culture supernatant of HepAD38 and purified by sucrose cushion ultracentrifugation. To confirm a successful HBV infection, we measured secretion of HBsAg and HBeAg on HepG2-NTCP-C4 following HBV infection. According to a published protocol (Iwamoto et al., PMID: 24342612), we pretreated HepG2-NTCP-C4 with 2.5% DMSO before infection and infected these hepatocytes with HBV virions in the presence of 4% PEG8000 and 2.5% DMSO. We collected culture supernatants at 1 to 9 days post infections (dpi) and measured viral HBsAg and HBeAg by Elecsys HBsAg and HBeAg (Cobas, Roche) assay. As shown in the revised Fig 3A-C, we successfully detected extracellular HBsAg and HBeAg upon HBV infection. In addition, intracellular LHBS and SHBS were detected by western blotting. Although SHBS expression was weak in this infectious model (in comparison to plasmid transfection), the expression of viral LHBS was clearly detected upon HBV infection. Using this infectious model, PKM2 activity was reduced by 30% following HBV infection on HepG2-NTCP-C4 (Revised Fig 3C). These data confirmed that PKM2 activity is reduced by HBV in an infectious model using HepG2-NTCP-C4.

**Revised Fig 3 A-C. HBV reduces PKM2 kinase activity and provokes aerobic glycolysis.** (A) HepG2-NTCP-C4 cells were infected with HBV virions at 1000 MOI in the presence of 4% PEG8000 and 2.5% DMSO. Secretions of viral HBsAg and HBeAg were measured in culture supernatants harvested at indicated time. Signal-to-cutoff (s/co) values of HBsAg and HBeAg were shown. (B) HepG2-NTCP-C4 cells were infected with HBV at 1000 MOI for 9 days and total cell lysates were harvested for the detection of LHBS, SHBS and PKM2 by western blotting. GAPDH expression was detected as an internal control. (C) HepG2-NTCP-C4 cells were infected with or without HBV at 1000 MOI for 9 days. Total cell lysates were collected for measuring PKM2 kinase activity. Relative PKM2 activities were shown after normalization with that of non-infected cells. All quantitative results were a summary of three repeats and data were displayed as mean ± standard error. *p<0.05 was calculated using Student's t test.

As suggested by the reviewer, we investigated the interaction between PKM2 and all envelope proteins. Although we have successfully detected extracellular HBsAg and HBeAg by the Elecsys assay on the infectious model of HepG2-NTCP-C4, the intracellular expression of SHBS was weaker than expected, as shown in the revised Fig 3B. Accordingly, it was not feasible to conduct protein immunoprecipitation of viral envelope proteins based on HBV-infected HepG2-NTCP-C4. As an alternative approach, we transfected HuH-7 cells with a plasmid carrying a complete HBV genome (pHBV3.6) and performed immunoprecipitation using monoclonal and polyclonal antibodies specific for HBS. As all three envelope proteins (L, M, and SHBS) contain the same C-terminus HBS region, immunoprecipitation with anti-HBS antibodies should pull-down all three envelope proteins at once. As shown in the revised Fig 2A, PKM2 was co-precipitated with viral envelope proteins by both monoclonal and polyclonal anti-HBs antibodies. To identify the interacting domain of the viral envelope protein responsible for PKM2 interaction, we generated expression constructs of LHBS, MHBS, SHBS, and PreS protein and tested their affinity with PKM2. As shown in the revised Fig. 2B, PKM2 interacted with LHBS, MHBS, and SHBS, but not PreS proteins. Together with the above results, we conclude that PKM2 interacts with all three envelope proteins of HBV.

Revised Fig 2. Identifying the minimal binding region of LHBS and PKM2. (A) Total cell lysates of HuH-7 cells transfected with pHBV3.6 plasmid were harvested for affinity immunoprecipitations using control immunoglobulin (IgG), monoclonal (mAb, clone 86H6) and polyclonal (pAb, Abnova) anti-HBS antibodies. The resulted immunoprecipitants were probed with anti-HBS mAb (86H6) and anti-PKM2 antibodies. Representative immunoblots were shown. (B) 293T cells were transiently transfected with expression constructs of HA-PKM2 and SNAP-tagged-LHBS, -MHBS, -SHBS, and -PreS, as indicated, for 2 days, and total cell lysates were harvested for affinity immunoprecipitation using a mouse anti-HA antibody. Representative immunoblots using anti-PKM2 and anti-SNAP antibodies were shown. (C) Immortalized hepatocytes stably expressing LHBS were transfected with expression constructs of HA-PKM2 encoding different truncation fragments as indicated. Total cell lysates were harvested at 2 days post transfection and subjected to affinity immunoprecipitation using anti-HA antibody. Representative immunoblots of HA-PKM2 and LHBS detected by anti-HA and anti-LHBS/PreS1 antibodies were shown. *denotes the high exposure image of the low molecular weight protein HA-367-476.

2. In the revised manuscript, we have provided detailed description about quantitation and statistical analysis in figure legends and the method section as appropriate. For the reviewer’s convenience, we have provided the revised paragraph of quantification and statistical analysis in the below section.

Revised manuscript page 23-24, line 541-552:

**Quantification and statistical analysis**

All experiments were repeated independently at least three times. Protein quantitations were done by detecting signal intensities of Western blots using ImageQuant LAS 4000 digital imaging system (GE Healthcare) and quantified using the accompanying ImageQuant TL software (GE Healthcare). Subtraction of background signals was done independently for each individual blot. All quantifications were performed within the linear range of the instruments in the absence of signal saturation. To control for possible variations in sample loading, protein signals were normalized to internal control signals of GAPDH, tubulin, or actin, as indicated, then calculated the fold changes between experimental and control groups. For PKM2 oligomerization analysis, the signal detected for the dimer fractions was normalized with the total PKM2 detected as monomer in the non-crosslinking counterpart. Statistical analysis was performed using Student’s t test. p<0.05 is considered significant. All data were displayed as mean ± standard error.

3. As suggested by the reviewer, we measured protein stabilities of LHBS, SHBS, HBcAg, and HBx in HuH-7 cells transfected with pHBV3.6. To investigate whether activation of PKM2 may affect general stability of HBV viral products, we treated cells simultaneously with cycloheximide and PKM2 activators and collected cell lysates at 0, 2, 4, 8, 12 and 14 hours. Our data indicated that general HBV viral protein stabilities were not significantly changed upon the activation of PKM2 by TEPP-46 and DASA-58. These new data are provided in the revised S2 Fig.

**Revised S2 Fig. Viral protein stabilities are not affected by PKM2 activators.** HuH-7 cells were transfected with pHBV3.6 for 24 hours and then treated simultaneously with 100 μg/ml cycloheximide, DMSO, 20 μM TEPP-46 or 50 μM DASA-58, as indicated. Total cell lysates were collected at indicated time points and subjected to western blotting using antibodies against LHBS/PreS1, SHBS, HBcAg, HBx, and GAPDH. Representative blots are shown.

4. As suggested by the reviewer, we measured secretion of HBV virions in HBV-infected HepG2-NTCP-C4 cells, with or without treatments of chemical PKM2 activators. The presence of HBV virions was measured by detecting HBV DNA in the culture supernatant using quantitative PCR. We found that HBV DNA was reduced by approximately 20% upon treatment of TEPP-46 and DASA-58. By statistical analysis of four independent experiments, only the reduction of HBV DNA upon TEPP-46 treatment was significant. This new result is provided in the revised Fig 6E.

**Revised Fig 6E. Activators of PKM2 diminished HBV biosynthesis.** (E) The production of virions in HBV-infected HepG2-NTCP-C4 cells, with or without treatments of chemical PKM2 activators were measured by detecting HBV DNA in the culture supernatant using quantitative PCR. All quantitative results were a summary of three repeats and data were displayed as mean ± standard error. *p<0.05 was calculated using Student's t test.

Reviewer #2: 1. Since the authors mapped the interaction domain on L to the S domain, would expression of S alone show the same effects on the cells as L? Is it possible the effects observed with overexpression of L was due to indirect consequences of L retaining in the cell, e.g., ER stress response?

2. Is it possible to further map the interacting region within the S domain? As S is normally rapidly secreted from the cell, S expression may not lead to high levels of intracellular S protein. However, it may be possible to express the interacting from S in the cell and see if it would have the same effects as L expression.

1. We thank the reviewer’s suggestion. To clarify whether expression of S alone has the same effect as L on modulating PKM2, we generated two HBV mutants of which the start codon of L on the PreS1 region and start codon of M on the PreS2 region were mutated by site-directed mutagenesis of pHBV3.6, i.e., a L-deficient mutant and a L/M-deficient mutant. Accordingly, these two HBV mutant constructs only allowed expressions of MHBS+SHBS, or SHBS alone on transfected hepatocytes. We found that PKM2 kinase activity was reduced in hepatocytes expressing MHBS+SHBS or SHBS alone. These new results indicated that expression of S alone was sufficient to reduce PKM2 activity in hepatocytes. These new data are now provided in the revised S1 Fig.

**Revised S1 Fig. Expressions of MHBS and SHBS reduced PKM2 activity.** (A) HuH-7 cells were transiently transfected with pHBV3.6 wild type (WT), start codon mutant on PreS1 (ΔL) and double start codon mutant on PreS1/S2 (ΔL/M) for 48 hours. Expressions of LHBS and SHBS were detected by Western blotting. (B) PKM2 kinase activity was measured on transfected hepatocytes at 48 hours post-transfection. The quantitative result was a summary of five repeats and data were displayed as mean ± standard error. n.s., not significant. * p<0.05 and ** p<0.01 were calculated using Student's t test.

2. To test whether PKM2 may interact with SHBS, we performed immunoprecipitations using monoclonal and polyclonal anti-HBS antibodies. As expected, PKM2 was co-precipitated with viral envelope proteins. These new data are provided in the revised Fig 2A. We consider it is not necessary to map the small interacting domain within the S coding region as all three viral envelope proteins (L, M, and SHBS) share the same C-terminus HBS region. In the revised Fig 2B, we show that PKM2 interacted with all three envelope proteins, but not PreS alone. Together with the above results, we conclude that PKM2 interacts with all three envelope proteins of HBV. For the convenience of the reviewer, we have provided new Fig 2 in the below section.

**Revised Fig 2. Identifying the minimal binding region of LHBS and PKM2.** (A) Total cell lysates of HuH-7 cells transfected with pHBV3.6 plasmid were harvested for affinity immunoprecipitations using control immunoglobulin (IgG), monoclonal (mAb, clone 86H6) and polyclonal (pAb, Abnova) anti-HBS antibodies. The resulted immunoprecipitants were probed with anti-HBS mAb (86H6) and anti-PKM2 antibodies. Representative immunoblots were shown. (B) 293T cells were transiently transfected with expression constructs of HA-PKM2 and SNAP-tagged-LHBS, -MHBS, -SHBS, and -PreS, as indicated, for 2 days, and total cell lysates were harvested for affinity immunoprecipitation using a mouse anti-HA antibody. Representative immunoblots using anti-PKM2 and anti-SNAP antibodies were shown. (C) Immortalized hepatocytes stably expressing LHBS were transfected with expression constructs of HA-PKM2 encoding different truncation fragments as indicated. Total cell lysates were harvested at 2 days post transfection and subjected to affinity immunoprecipitation using anti-HA antibody. Representative immunoblots of HA-PKM2 and LHBS detected by anti-HA and anti-LHBS/PreS1 antibodies were shown. *denotes the high exposure image of the low molecular weight protein HA-367-476.

3. As HBV infection systems are now readily available, e.g., HepG2-NTCP or better, primary human hepatocytes, the authors need to verify the effects of L overexpression using these more authentic infection systems.

As suggested by the reviewer, we have tested HBV infection on primary human hepatocytes (a review article detailed the comparison of different cell models was included in the reference 19 (Hu et al., PMID: 30243619). The infectious HBV virions were collected from the culture supernatant of HepAD38 and purified by sucrose cushion ultracentrifugation. We obtained two different batches of primary human hepatocytes from Gibco (LOT #HU1924 and LOT #HU8287), and only one batch of primary hepatocytes (LOT#HU8287) had reached 60% of overall cell viability after thawing. We successfully expanded and cultivated this batch of primary human hepatocytes and performed an infection assay using a published protocol (Gripon et al., PMID: 8421898). Accordingly, we pretreated primary hepatocytes with 2.5% DMSO before infection and infected these hepatocytes with HBV virions at 5000 MOI in the presence of 4% PEG8000 and 2.5% DMSO. We had collected culture supernatants at 1 to 9 day post infections (dpi) and measured viral HBsAg and HBeAg by Elecsys HBsAg and HBeAg (Cobas, Roche) assay. Unfortunately, those results were not satisfying. Using data obtained at 7 dpi as a representative, we only detected a low amount of HBsAg secretion upon HBV infection, with a signal-to-cutoff values below 3 (Response Fig 1 provided in below, this result is not included in the revised manuscript, provided only for the review). In addition, the HBeAg was not detected in all groups, indicating the level of HBeAg was lower than the Cobas assay's detection limit. Therefore, we could not work on primary human hepatocytes.

As an alternative approach, we worked with HepG2-NTCP-C4 cells infected with HBV and had successfully detected significant levels of HBsAg and HBeAg in culture supernatants. By working with HepG2-NTCP-C4 cells, we now have demonstrated that PKM2 activity was reduced by approximately 30% upon HBV infection. These new data are included in the revised manuscript Fig 3A and 3C. We noted that the reduction of PKM2 activity was more intense in the LHBS-expressing immortalized hepatocytes than that in HBV-infected HepG2-NTCP-C4 cells. We consider this result can be explained by the poor infection efficiency of HBV, i.e., not all HepG2-NTCP-C4 cells were infected by HBV, even a high viral load was used for infection. This notion has been mentioned in the discussion section.

We hope the reviewer could kindly accept these results, as the infectious model using authentic HBV on primary human hepatocytes was limited by the poor infection efficiency. And we have successfully demonstrated the impact of HBV on modulating PKM2 activity with HepG2-NTCP-C4 cells. These new evidences should provide sufficient support for our conclusion.

The following results are provided for the reviewer, but not included in the revised manuscript.

**Response Fig 1 (not included in the manuscript). Comparison of extracellular HBsAg and HBeAg in culture media collected from HBV-infected primary human hepatocytes (PHH) and HepG2-NTCP-C4 cells.** PHHs were infected with HBV virions at 5000 MOI and HepG2-NTCP-C4 were infected at 1000 MOI, in the presence of 4% PEG8000 and 2.5% DMSO for 24 hours. Inoculum virus was washed away at 24 hours post-infection and culture supernatants were collected at 1-9 days. The figure showed representative quantitation results of extracellular HBsAg (left) and HBeAg (right) obtained at day 7 post infection. The quantitative result was a summary of two repeats (PHH) and three repeats (HepG2-NTCP-C4) and data were displayed as mean ± standard error.

4. What are the effects of HBV on PKM2, and PKM2 on HBV, in the 3-dimensional agarose culture vs. the traditional 2-dimensional cell culture? Can any of those effects be correlated with the effects on cell proliferation?

We thank the reviewer for pointing this out. In this study, we showed that expression of LHBS supported colony formation of immortalized hepatocytes in soft agar and the restoration of PKM2 activity by TEPP-46 or DASA-58 could significantly reduce cell proliferation in the soft agar (Revised Fig 9). Noted that TEPP-46 did not influence hepatocyte proliferation on the traditional 2-dimensional culture. We suspect that cell growth in 3-dimensional soft agar can be a stress condition for immortalized hepatocytes, as the parental immortalized hepatocytes grown well in the 2-dimensional culture (data not shown) but failed to form colony formation in the soft agar (Revised Fig 9A). Therefore, the expression of LHBS was essential for the induction of anchorage-independent growth of immortalized hepatocytes. LHBS has been long been considered as a viral oncoprotein in previous reports (Li et al., PMID: 29862509; and a recent review by Lin et al., PMID: 32911838). Transgenic mice expression LHBS developed liver dysplastic changes and promoted hepatocarcinogenesis (Chisari et al., PMID: 7875658). Accordingly, we suggest that although the induction of metabolic switch via modulating PKM2 activity may contribute to cell adaptation in the 3-dimensional culture, the oncogenic property of viral surface antigen, especially the LHBS, was critical for the ultimate hepatocarcinogenesis. To clarify this issue, we have provided an additional paragraph in the discussion (revised manuscript page 14, line 320-327).

**Revised Fig 9. PKM2 activators suppressed LHBS-mediated oncogenesis on immortalized hepatocytes.** (A) Colony formation assay was performed with control and LHBS-positive immortalized hepatocytes for 3 weeks in soft agar plates. Representative plate images are shown. No colony formation was detected in control cells. Quantitation of colony numbers were provided in the right panel. (B) LHBS-positive hepatocytes were treated with DMSO or 20 μΜ TEPP-46 for 24 hours and cell lysates were harvested for measuring PKM2 activity. (C) Cell viability was measured in LHBS-positive hepatocytes 48 hours after treatments with DMSO or 20 μΜ TEPP-46. (D) Representative colony formation images of control, and LHBS-positive hepatocytes treated with DMSO or 20 μΜ TEPP-46 were shown. Quantitation results of colony numbers were shown on the right panel. (E) Representative colony formation images of control, and LHBS-positive hepatocytes treated with DMSO or 50 μΜ DASA-58 were shown. Quantitation results of colony numbers were shown on the right panel. All quantitative results were a summary of three repeats and data were displayed as mean ± standard error. *p<0.05 was calculated using Student's t test.

Reviewer #3: 1. It is not clear whether N-terminal SNAP tag interferes with the membrane topology of LHBs, MHBs and SHBs proteins? It appears that all the experiments presented in Figs 1 to 3 used SNAP-tagged HBV envelope proteins. Because SNAP tag is a 182 residues polypeptide and might interfere with the structure and function of HBV envelope proteins, it is important to demonstrate PKM2 interacts with native L, M and S proteins in the context of HBV replication.

We thank the reviewer for pointing this out. To clarify if native envelope proteins interact with PKM2, we performed immunoprecipitation of viral envelope proteins in HuH-7 cells transfected with pHBV3.6 plasmid. We confirmed that PKM2 was co-precipitated with native viral envelope proteins under affinity immunoprecipitations using either monoclonal or polyclonal antibodies against HBS. These new data are provided in the revised Fig 2.

**Revised Fig 2. Identifying the minimal binding region of LHBS and PKM2.** (A) Total cell lysates of HuH-7 cells transfected with pHBV3.6 plasmid were harvested for affinity immunoprecipitations using control immunoglobulin (IgG), monoclonal (mAb, clone 86H6) and polyclonal (pAb, Abnova) anti-HBS antibodies. The resulted immunoprecipitants were probed with anti-HBS mAb (86H6) and anti-PKM2 antibodies. Representative immunoblots were shown. (B) 293T cells were transiently transfected with expression constructs of HA-PKM2 and SNAP-tagged-LHBS, -MHBS, -SHBS, and -PreS, as indicated, for 2 days, and total cell lysates were harvested for affinity immunoprecipitation using a mouse anti-HA antibody. Representative immunoblots using anti-PKM2 and anti-SNAP antibodies were shown. (C) Immortalized hepatocytes stably expressing LHBS were transfected with expression constructs of HA-PKM2 encoding different truncation fragments as indicated. Total cell lysates were harvested at 2 days post transfection and subjected to affinity immunoprecipitation using anti-HA antibody. Representative immunoblots of HA-PKM2 and LHBS detected by anti-HA and anti-LHBS/PreS1 antibodies were shown. *denotes the high exposure image of the low molecular weight protein HA-367-476.

2. Because all the three envelope proteins, L, M and S, interact with PKM2, does the expression of MHBs or SHBs also inhibit the enzymatic activity of PKM2? It is rather striking that expression of SNAP-LHBs alone reduced PKM2 activity by 86%, but infection of Huh7 cells with Ad-HBV-WT only inhibited PKM2 activity by 13%, suggesting a very weak effect in the context of HBV replication.

We thank the reviewer for pointing this out. In this revision, we performed new experiments to clarify this issue. To avoid a potential artifact elicited by overexpression of SNAP-tagged proteins, we have generated start-codon mutants of PreS1 and PreS2 open reading frames of envelope proteins on the basis of pHBV3.6 vector. The construct labeled as ∆L expressed M and S proteins upon transfection into HuH-7 cells and the ∆L/M construct expressed only S proteins. We found that PKM2 activity was reduced by approximately 15% in cells transfected with either wild type, ∆L, or ∆L/M constructs. Accordingly, MHBS and SHBS were able to modulate PKM2 activity in hepatocytes. These new data are provided in the revised S1 Fig in this revision. To strengthen our conclusion, we also provide new data based on HBV-infected HepG2-NTCP-C4 cells. In this infectious model, we show that HBV infection reduced PKM2 activity on HepG2-NTCP-C4 cells. This new data is provided in the revised Fig 3C.

Next, we consider it is reasonable to see PKM2 activity more profoundly reduced in cells stably expressed SNAP-LHBS than cells infected with Ad-HBV or transfected with pHBV3.6 plasmid. As the reduction of PKM2 activity relied on the interaction with viral envelope proteins, immortalized hepatocytes with stable expression of LHBS would theoretically impact PKM2 in the whole cell populations. In cases of infections with Ad-HBV or HBV or transfection with pHBV3.6, only a certain percentage of cells were successfully infected or transfected. Our transfection efficiency was approximately 25% and the infection efficiency of Ad-HBV (with 100 MOI) was around 15%. Therefore, modulation of PKM2 activity may rely on the expression of viral envelope proteins in the cell population. This notion has now been discussed in the discussion (revised manuscript page 13, line 291-299).

3. In evaluating the relationship between PKM2 activity and HBV replication (Figs. 5 to 8), only viral protein expression/secretion was measured. In order to obtain further mechanistic insights, HBV RNA and core DNA should also be determined. More importantly, the effects of HBV on cellular glycolysis and impact of glycolysis on HBV replication should be further validated in HBV infected primary human hepatocytes, because the glycolysis pathway of hepatoma cells (HepG2 and Huh7) may be altered.

We thank the reviewer for the suggestions. We have measured levels of PreS1, SHBS, and core RNA in pHBV3.6-transfected HuH-7 cells with or without treatments of PKM2 activators. We found that viral RNAs were not reduced by PKM2 activation. Therefore, the reduction of HBV biosynthesis was not likely due to the reduction of viral transcription. These new data are now provided in the revised S3 Fig. In this study, we propose that HBV biosynthesis is supported by the induction of metabolic switch to aerobic glycolysis, which provokes de novo synthesis via pentose phosphate pathway. In the revised manuscript, we provided evidence that 6-PGA, an indicator of the pentose phosphate pathway, was reduced following restoration of PKM2 activity (revised Fig 6D). Therefore, the induction of aerobic glycolysis did not impact on HBV replication, rather, on the de novo biosynthesis of viral proteins such as envelope and core proteins.

**Revised S3 Fig. The impact of PKM2 activation on viral transcription.** HuH-7 cells were transfected with pHBV3.6 for 24 hours and then treated with DMSO, 20 μΜ TEPP-46 or 50 μΜ DASA-58 for additional 24 hours. Total cell RNA was extracted and subjected to reverse transcription quantitative PCR using primers specific for SHBS, PreS1 and HBcAg coding sequences. The quantitative results were a summary of three independent repeats and data were displayed as mean ± standard error.

Finally, as suggested by the reviewer, we examined HBV infection on two different batches of primary human hepatocytes. The results were inconclusive as the expression of HBsAg and HBeAg on primary hepatocytes was much lower than HepG2-NTCP-C4 cells (Data provided in the below Figure). As mentioned by the reviewer, glycolysis pathway can be changed in HepG2 or HuH-7 cells. We argue that glucose metabolism is also changed on primary human hepatocytes, as treatment with 2.5% DMSO is generally required to enhance HBV infection on human primary hepatocytes and HepG2-NTCP-C4. Despite the potential caveat of altered glucose metabolism, we had successfully worked on HepG2-NTCP-C4 cells and demonstrated that PKM2 modulation has an impact on HBV biosynthesis, as shown in the revised Fig 3C and 6E. Together with all data supported in this study, these new evidences strongly support our conclusion that PKM2 is involved in modulating HBV biosynthesis.

**Response Fig 1 (not included in the manuscript). Comparison of extracellular HBsAg and HBeAg in culture media collected from HBV-infected primary human hepatocytes (PHH) and HepG2-NTCP-C4 cells.** PHHs were infected with HBV virions at 5000 MOI and HepG2-NTCP-C4 were infected at 1000 MOI, in the presence of 4% PEG8000 and 2.5% DMSO for 24 hours. Inoculum virus was washed away at 24 hours post-infection and culture supernatants were collected at 1-9 days. The figure showed representative quantitation results of extracellular HBsAg (left) and HBeAg (right) obtained at day 7 post infection. The quantitative result was a summary of two repeats (PHH) and three repeats (HepG2-NTCP-C4) and data were displayed as mean ± standard error.

4. If LHBs interaction with PKM2 underlines HBV oncogenic activity, it can be anticipated that MHBs and SHBs should have a similar effect? Is this true?

We consider LHBS is a stronger viral oncogene than MHBS and SHBS. Although PKM2 interacts with all three envelope proteins, the induction of PKM2-dependent metabolic change alone is not sufficient to support hepatocarcinogenesis. LHBS has been long been considered as a viral oncoprotein in previous reports (Li et al., PMID: 29862509; and a recent review by Lin et al., PMID: 32911838). Transgenic mice expression LHBS developed liver dysplastic changes and promoted hepatocarcinogenesis (Chisari et al., PMID: 7875658). In contrast, transgenic mice expressing only SHBS did not cause hepatocyte dysplastic changes or other oncogenic effects (Francis Chisari, PMID: 7557887). Therefore, we suggest that although the induction of metabolic switch via modulating PKM2 activity may contribute to cell adaptation in the 3-dimensional culture, the oncogenic property of viral surface antigen, especially the LHBS, was critical for the ultimate hepatocarcinogenesis. To clarify this issue, we have provided an additional paragraph in the discussion (revised manuscript page 14, line 320-327).

**Part III – Minor Issues: Editorial and Data Presentation Modifications**
Please use this section for editorial suggestions as well as relatively minor modifications of existing data that would enhance clarity.

Reviewer #1: (No Response)

Reviewer #2: 1. Fig. 1B: Images from a single cell are not acceptable! Quantitative results are needed.

We thank the reviewer for pointing this out. In the revised Fig 1B, we use proximity ligation assay (PLA) to detect intracellular interaction between LHBS and PKM2 on immortalized hepatocytes. In this case, the control hepatocyte expressing only SNAP protein was used as the negative control. The PLA was performed with incubation of primary antibodies specific for LHBS/PreS1 and PKM2, followed by fluorescent-labeled secondary antibodies, and detection of PLA signals with specific PLA probes. Under the same experimental procedures and imaging conditions, we detected no PLA signal on the control lines, and all LHBS-positive cells were detected with dot-like PLA signal in the cytoplasm (Revised Fig 1B, right panels). As these were all-or-none experimental results, we consider simply showing the quantitation result may not be sufficient to reveal the spatial information for the PKM2-LHBS interaction. In the revised manuscript, we have provided multiple representative images of LHBS-positive cells, reporting intracellular distribution of LHBS, PKM2, and where two proteins interacted with each other in the cytoplasm.

**Revised Fig 1. PKM2 is a binding partner of viral LHBS.** (B) Visualization of PKM2-LHBS interactions in immortalized hepatocytes by proximity ligation assay (PLA). Control and LHBS-positive cells were stained with primary antibodies against LHBS/PreS1 and PKM2, followed by incubation with Duolink PLA probes detecting LHBS/PKM2 interactions and fluorescent labeled secondary antibodies. The cell nuclei were stained with DAPI. Three representative images were shown for each category. Scale bar denotes 10 µm.

2. Line 336: Ad-HBV-KO needs to be described more clearly.

We thank the reviewer’s suggestion. We have updated the reagent information in the revised manuscript as appropriate (revised manuscript page 17, line 394-401).

**Infection with Ad-HBV**

Ad-HBV-KO and Ad-HBV-WT were the kind gifts from Dr. Li-Rung Huang (National Health Research Institutes, Taiwan) and produced according to a previous publication [21]. Specifically, Ad-HBV was produced by HEK293 cell transfection with adenovirus package plasmids. The HBV1.3 and HBV1.3 KO genomes were replaced into the E1 region of adeno-virus (Ad5ΔE1/E3) backbone plasmid. HBV1.3 KO contained premature stop codons in all HBV open reading frames. For the infection with Ad-HBV, HuH-7 and HepG2 cells were seeded in 6 cm dishes overnight and infected with Ad-HBV-KO or Ad-HBV-WT at indicated MOI of 0 to 200 GEq/cell for 2 days.

3. Line 358-359: These Abs (for L, S, X) should be described in more details, or if published, relevant references need to be cited.

Mouse anti-LHBS/PreS1 (7H11), mouse anti-SHBS (86H6), and mouse anti-HBx (20F3) were kindly provided by Professor Ning-Shao Xia (Xiamen University, China) (Gao et al., PMID: 28521640). In addition, we also updated all antibody information in the revised materials and methods, in a new section labeled as “Antibodies”.

4. Line 390: More details are needed for the PLA assay - probe sequences, Abs used, assay conditions, etc.

We have provided detailed information for the PLA assay in a new section on the method section (page 20-21, line 463-477).

**Proximity ligation assay**

Duolink In Situ Red Starter Kit Mouse/Rabbit (DUO92101) was purchased from Sigma-Aldrich and the assay followed Duolink PLA Fluorescence Protocol provided from the manufacturer. In brief, cells were seeded on collagen-coated coverslips, washed with PBS, and then fixed with 4% formaldehyde/PBS at room temperature for 10 minutes. The coverslips were treated with Duolink blocking solution at 37℃ for 1 hour then incubated with mouse anti-LHBS/PreS1 (7H11) and rabbit anti-PKM2 antibodies for another 1 hour. The coverslips were washed three times with PBS, and applied PLA probes (anti-rabbit PLUS and anti-mouse MINUS) at 37°C for 1 hour and continued with probe ligation and amplification according to manufacturer’s instruction. After signal amplification of PLA signals, the slides were incubated with donkey anti-mouse conjugated with AlexaFluor-647 and donkey anti-rabbit conjugated with AlexaFluor-488 for 1 hour, washed three times in PBS-0.05% Tween20, and mounted with DAPI-containing mounting medium. The fluorescence cell images were taken by Leica DMI6000 inverted microscope (Leica Microsystems) and an Andor Luca R EMCCD camera (Andor Technology). All images were acquired and processed with MetaMorph software (Molecular Devices).

Reviewer #3: No.

PLOS authors have the option to publish the peer review history of their article ([what does this mean?](https://journals.plos.org/plospathogens/s/editorial-and-peer-review-process#loc-peer-review-history)). If published, this will include your full peer review and any attached files.


If you choose “no”, your identity will remain anonymous but your review may still be made public.


**Do you want your identity to be public for this peer review?** For information about this choice, including consent withdrawal, please see our [Privacy Policy](https://www.plos.org/privacy-policy).

Reviewer #1: No

Reviewer #2: No

Reviewer #3: No
