# Supplementary material for: Aerobic glycolysis supports hepatitis B virus protein synthesis through interaction between viral surface antigen and pyruvate kinase isoform M2
Source: PLoS Pathog. 2021 Mar 15;17(3):e1008866. doi: 10.1371/journal.ppat.1008866 (PMC8009439; doi:10.1371/journal.ppat.1008866)
Supplement: Attachment — Submitted filename: Response to Reviewers.docx [file ppat.1008866.s007.docx]

Lily Hui-Ching Wang

Associate Professor

Institute of Molecular and Cellular Biology

National Tsing Hua University

Hsinchu, Taiwan

Tel: +886-35742069

lilywang@life.nthu.edu.tw

February 24, 2021

Dear editor,

Thank you very much for your positive response for our work. We have modified the manuscript according to the review recommendations.

In the revised manuscript, we made two major changes as summarized in below. Detailed responses to the review comments are also provided in the next page.

1. We revise the term “viral biosynthesis” to “viral protein synthesis” in the manuscript as appropriate, including title, abstract, and the main text. These changes are highlighted in the provided highlighted version of the manuscript.
2. We provide an additional paragraph to help interpret why activation of PKM2 selectively reduced viral protein synthesis in the context of HBV. This paragraph is now included in the revised discussion.

We appreciate our reviewers' effort to improve our manuscript and the editor's understanding of the challenges in our revision experiments, which gave us sufficient time to accomplish this work. Finally, we trust our finding can be of interest to the readers of *PLOS Pathogens* and thus look forward to your acceptance.

Yours sincerely,

Lily Wang

Associate Professor

Institute of Molecular and Cellular Biology

National Tsing Hua University

Hsinchu, Taiwan

Tel: +886-35742069

lilywang@life.nthu.edu.tw

Reviewer's Responses to Questions

**Part I - Summary**
Please use this section to discuss strengths/weaknesses of study, novelty/significance, general execution and scholarship.

Reviewer #1: In the revised version of the manuscript, the authors have satisfactorily answered the main questions of this reviewer. They have performed a considerable number of experiments to obtain a better quantification in their study, and have modified the text accordingly.

Thank you for the positive feedback.

Reviewer #2: The authors have adequately addressed my previous concerns.

Thank you for the positive feedback.

Reviewer #3: The authors made great efforts to address my concerns on the previous version. The manuscript is significantly improved. The following minor points are intended to help further improve the manuscript.

1. The word “viral biosynthesis” or “de novo biosynthesis” does not specifically refer to any specific viral replication step or biosynthesis event. As your results seem to indicate that activation of PMK2 selectively inhibit the synthesis of viral envelope proteins, core/pre-core (HBeAg) proteins, but not HBx and multiple cellular proteins tested, I would like to suggest the authors should consider to more specifically state that aerobic glycolysis modulates viral protein synthesis (or translation), instead of “viral biosynthesis”.

We thank the reviewer for helping to improve our manuscript. We agree with the reviewer that the word “viral protein synthesis” may more specifically state our finding than “viral biosynthesis”, as we showed that either viral protein stability (S2 Fig) or viral transcription (S3 Fig) were not affected by PKM2 activation.

In this revision, we have revised the term “biosynthesis” to “protein synthesis” in the manuscript as appropriate. These modifications are highlighted in the highlighted version.

2. The authors stated that activation of PMK2 selectively reduces the levels of viral proteins via pentose phosphate pathway. Please provide more clear interpretation. It will be great if the authors can speculate how the viral protein synthesis (translation) is selectively modulated by aerobic glycolysis metabolism in Discussion.

We thank the reviewer’s suggestion. We have included an additional paragraph to help interpret why activation of PKM2 selectively reduced viral protein synthesis in the context of HBV.

For the reviewer’s convenience, we have provided the revised paragraph below.

Page 13, line 306:

Why the protein synthesis of HBV is more sensitive to metabolic rewiring than the host machinery is an interesting question to be clarified. As viral replication is a process involving an extremely high turnover rate, a shortage in supply of amino acid precursors may respond quickly to delay viral protein synthesis. In this regard, hijacking the metabolic supplies of macromolecules from the host is a common strategy used by viruses for reproduction [23, 27]. The pentose phosphate pathway is a fundamental metabolism component that provides precursors for nucleotide and amino acid biosynthesis, thus serving as ideal support to fuel viral reproduction. We suspect that HBV hijacks newly synthesized building blocks, probably coming from the pentose phosphate pathway, for the demand of viral reproduction at high speed. This notion explains why protein synthesis of HBV is more sensitive to metabolic rewiring than that of general host proteins, as also supported by our observation that 6-PGA, a pentose phosphate pathway product, was reduced upon PKM2 activation (Fig 6D). In short, our results imply that an efficient HBV reproduction relies on sufficient supports from the pentose phosphate pathway and aerobic glycolysis.

3. Although the authors more carefully measured the effects of PMK2 activation on the levels of three viral proteins, the effects on viral DNA replication (intracellular core DNA), as requested by multiple reviewers, have not been measured in either pHBV3.6 transfected Huh7 cells or HBV infected HepG2-NTCP cells. Because of the unique translation mechanism of viral DNA polymerase, it will be very interesting to know if its synthesis is differentially regulated. Obviously, if the synthesis of DNA polymerase is inhibited, viral DNA synthesis should be consequentially reduced. Therefore, this assay could be an indirect measurement of aerobic glycolysis regulation of DNA pol translation.

We thank the reviewer's comments. We have shown that the viral core RNA level was not affected upon PKM2 activation, implying that aerobic glycolysis does not affect general viral transcription. As viral core and polymerase are translated from the same viral pregenomic RNA, it is plausible that viral polymerase translation is also sensitive to aerobic glycolysis. However, translation initiation of viral polymerase is unique. Ribosomes first scan from the encapsidated 5'end of pregenomic RNA, skip the four upstream AUG codons by leaky scanning, and then translate and terminate a short J open reading frame then initiate polymerase translation. We want to emphasize that viral polymerase's translation elongation process is no different from other viral products. We agree with the reviewer that the impact of aerobic glycolysis on the overall synthesis of viral polymerase is an interesting topic to be explored. We hope to clarify this issue in future studies.

**Part II – Major Issues: Key Experiments Required for Acceptance**
Please use this section to detail the key new experiments or modifications of existing experiments that should be absolutely required to validate study conclusions.

Generally, there should be no more than 3 such required experiments or major modifications for a "Major Revision" recommendation. If more than 3 experiments are necessary to validate the study conclusions, then you are encouraged to recommend "Reject".

Reviewer #1: None

Reviewer #2: (No Response)

Reviewer #3: No.

**Part III – Minor Issues: Editorial and Data Presentation Modifications**
Please use this section for editorial suggestions as well as relatively minor modifications of existing data that would enhance clarity.

Reviewer #1: None

Reviewer #2: (No Response)

Reviewer #3: No.

PLOS authors have the option to publish the peer review history of their article ([what does this mean?](https://journals.plos.org/plospathogens/s/editorial-and-peer-review-process#loc-peer-review-history)). If published, this will include your full peer review and any attached files.


If you choose “no”, your identity will remain anonymous but your review may still be made public.


**Do you want your identity to be public for this peer review?** For information about this choice, including consent withdrawal, please see our [Privacy Policy](https://www.plos.org/privacy-policy).

Reviewer #1: No

Reviewer #2: No

Reviewer #3: No
